# Melanin-based plumage coloration and melanin content in organs in the barn owl

**DOI:** 10.1007/s10336-023-02137-w

**Published:** 2023-12-26

**Authors:** Alexandre Roulin, Sylvain Dubey, Shosuke Ito, Kazumasa Wakamatsu

**Affiliations:** 1 Department of Ecology and Evolution, University of Lausanne, Biophore Building, CH-1015 Lausanne, Switzerland; 2 HW Romandie SA, Avenue Des Alpes 25, CH-1820 Montreux, Switzerland; 3 Institute for Melanin Chemistry, Fujita Health University, Toyoake, Aichi 470-1192 Japan

**Keywords:** Color polymorphism, Melanin, Natural selection, Sexual selection, *Tyto alba*

## Abstract

Although the evolutionary ecology of melanin pigments and melanin-based coloration has been studied in great details, particularly in birds, little is known about the function of melanin stored inside the body. In the barn owl *Tyto alba*, in which individuals vary in the degree of reddish pheomelanin-based coloration and in the size of black eumelanic feather spots, we measured the concentration in melanin pigments in seven organs. The eyes had by far the most melanin then the skin, pectoral muscle, heart, liver, trachea, and uropygial gland. The concentration in eumelanin was not necessarily correlated with the concentration in pheomelanin suggesting that their production can be regulated independently from each other. Redder barn owls had more pheomelanin in the skin and uropygial gland than white owls, while owls displaying larger black feather spots had more eumelanin in the skin than small-spotted owls. More data are required to evaluate whether melanin-based traits can evolve as an indirect response to selection exerted on melanin deposition in organs.

## Abstract (de)

**Melaninbasierte Gefiederfärbung und Melaningehalt in Organen der Schleiereule**

Obwohl die evolutionäre Ökologie von Melaninpigmenten und melaninbasierter Färbung, insbesondere bei Vögeln, sehr detailliert untersucht wurde, ist nur wenig über die Funktion des im Körper gespeicherten Melanins bekannt. Bei der Schleiereule Tyto alba, bei der sich die Individuen im Grad der rötlichen Färbung auf Phäomelaninbasis und in der Größe der schwarzen Federflecken unterscheiden, haben wir die Konzentration der Melaninpigmente in sieben Organen gemessen. Die Augen wiesen bei weitem das meiste Melanin auf, gefolgt von der Haut, dem Brustmuskel, dem Herzen, der Leber, der Luftröhre und der Bürzeldrüse. Die Eumelaninkonzentration korrelierte nicht unbedingt mit der Phäomelaninkonzentration, was darauf hindeutet, dass ihre Produktion unabhängig voneinander reguliert werden kann. Rötliche Schleiereulen hatten mehr Phäomelanin in der Haut und der Bürzeldrüse als weiße Eulen, während Eulen mit größeren schwarzen Federflecken mehr Eumelanin in der Haut aufwiesen als kleinfleckige Eulen. Es sind weitere Daten erforderlich, um zu beurteilen, ob sich melaninbasierte Merkmale als indirekte Reaktion auf die Selektion, die auf die Melaninablagerung in den Organen ausgeübt wird, weiterentwickeln können.

## Introduction

In many birds, variation in the degree of blackish and reddish colorations is due to differential deposition of black eumelanin and reddish pheomelanin pigments, respectively (Ito and Wakamatsu 2003). Melanin-based colorations are used as mate choice criteria (Roulin 2016), play important roles in camouflage (Merilaita et al. 2017) and accumulate warmth and protect the body against various sources of stress including physical abrasion, parasites, ultraviolet, and free radicals (Bonser 1996; Clusella-Trullas et al. 2008; Gunderson et al. 2008; Meredith and Sarna 2006). Given the numerous functions of melanin and the pleiotropic effects of melanogenic genes (Ducrest et al. 2008), dark and light conspecifics can maximize their fitness in different habitats so that the sign and magnitude of selection exerted on melanin-based coloration are species- and trait-specific (Meunier et al. 2011).

Although recognized as important, less information has been published on the amount of melanin stored in internal organs not only in birds but also in other vertebrates. The available data indicate that melanin protects organs and the integument against the same sources of stress (Dubey and Roulin 2014; McNamara et al. 2021). It can also alter cellular messaging pathways and regulate gene expression (Pavan and Sturm 2019; Zhou et al. 2021). Melanin has been found to play a role in protecting against oxidative damage to tissues, organs, and cells. These functions may be beneficial for protecting organs and tissues from damages caused by various external sources such as the sun’s UV radiation to which mammalian species are often exposed (Meredith and Sarna 2006; d’Ischia et al. 2015). Additionally, melanin influences physiological processes including cell differentiation, pain perception, and blood pressure regulation (Bennett 1989; Mosley et al. 2000; Robinson et al. 2021; El-Naggar and Saber 2022). Melanin provides protection against harmful viruses and bacteria by scavenging free radicals generated from metal ions and other toxic species and by regulating inflammation (ElObeid et al. 2017; Guo et al. 2023). Melanin plays an important role in maintaining the normal structure and function of nerve cells, which is critical for the normal functioning of many organs (Tigran 2015; Chow et al. 2022). Finally, melanin is an important molecule to protect from heat stress that can damage internal organs (Zhang et al. 2023), which might be relevant to birds that need to keep their body temperature constant. It should be clarified that a similar response in epidermal/dermal tissues of birds (skin and feathers) is possible given that birds are also endotherms.

Under these conditions, we can predict that the amount of melanin deposited in feathers, hair, skin, and cuticle could be positively correlated with the amount of melanin produced in internal organs. This prediction would imply that integumental pigmentation could signal the extent to which internal melanin might protect organs. In other words, by displaying a melanic plumage, individuals could signal to potential partners and competitors their high quality because their organs are protected by melanin in the face of environmental stressful factors. Therefore, selection acting on the melanin-based coloration of the external body surface would affect the evolution of the amount of melanin stored inside organs. The opposite conclusion would also apply, namely that darker colorations of the external body surface could evolve as an indirect response to selection exerted on internal organs to be protected by melanin.

**Fig. 1:**
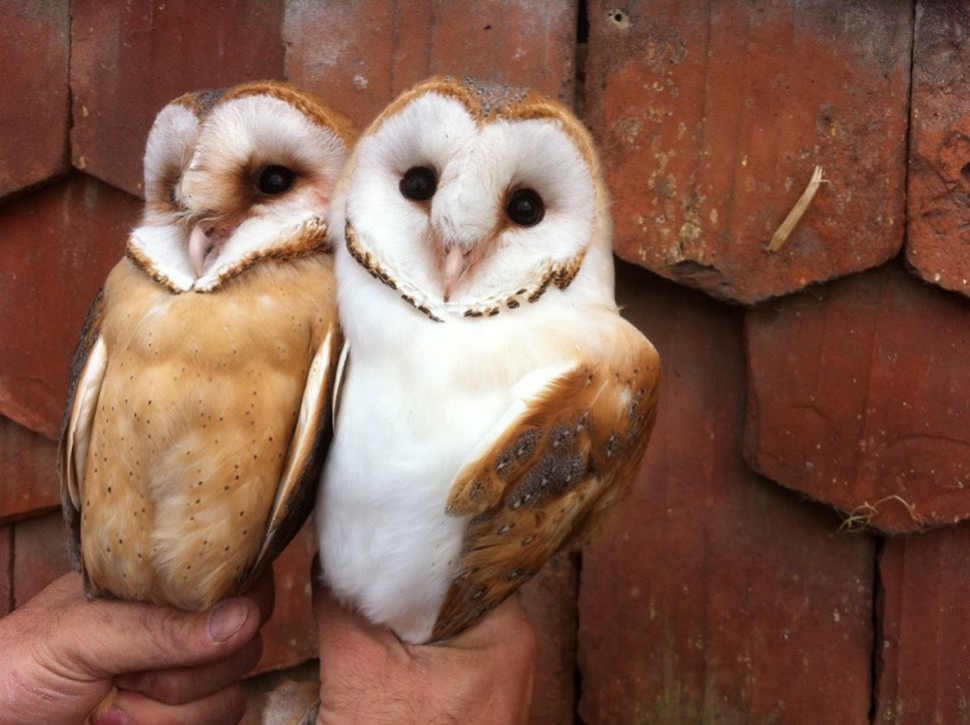
A dark reddish barn owl nestling displaying many black spots and a white and immaculate sibling. @ Alexandre Roulin

Given the paucity of information about internal melanin, descriptive studies identifying potential correlations between the concentration of melanin pigments in the integuments and internal organs are required before engaging in more detailed research. We performed such a study in the barn owl *Tyto alba*, a species showing pronounced variation in melanin-based plumage traits (Fig. 1). The body underside varies from white to reddish brown, because of differential deposition of pheomelanin pigments (Roulin et al. 2008). The plumage also varies from immaculate to marked with black spots of varying sizes, because of differential deposition of eumelanin pigments. While reddish and white barn owls have a slightly different diet (Roulin 2004a) and are adapted to different environmental conditions (Dreiss et al. 2012), variation in the size of black feather spots is mainly related to the capacity to resist a large range of environmental stressful factors (Roulin and Ducrest 2011). This raises the hypothesis that some of the reported associations between plumage spottiness and stress resistance might be due to melanin deposited in internal organs.

To perform the first evaluation of the hypothesis that melanin-based coloration is a signal of the ability of internal organs to be stress-resistant thanks to the deposition of melanin pigments inside them, we collected 31 dead cadavers along roads in France. We took a sample of the eyes, heart, liver, muscle, skin, trachea, and uropygial gland for which we measured the melanin content. We collected those organs as they could be easily extracted. Using this data set, we first determined which organ has the most internal melanin. Second, we investigated whether the melanin content in one organ is correlated with the melanin content in another organ and whether the eumelanin and pheomelanin content is correlated within each organ. Third, we tested for sexual dimorphism in the amount of melanin inside organs. Fourth, we examined whether the eumelanin content in organs is correlated with the size of black eumelanic feather spots and whether the pheomelanin content in organs is correlated with the degree of reddish pheomelanin-based plumage coloration.

Although we measured internal melanin in a limited number of individuals, we performed several statistical analyses at the risk of increasing type II errors (i.e., retaining a false hypothesis). Our goal is indeed to explore the data as much as possible to stimulate and guide further projects considering melanin content of internal organs, a field that is still in its infancy in ornithology.

## Methods

### Collecting internal organs

From 2014 to 2017, the “Association CHENE” (https://associationchene.com) collected dead barn owls along roads in Normandy, France. All cadavers were collected except the ones that were of poor quality (i.e., clearly destroyed). All cadavers stayed a short amount of time on the roads given that they were regularly monitored by people from the “Association CHENE”. Immediately upon collection, they were kept in a freezer, all under the same conditions. On 28 November 2017, all cadavers were thawed, and we collected aliquots of the eyes, heart, liver, muscle, skin, trachea, and uropygial gland were collected and stored in 30% ethanol before measuring melanin contents in March 2018. The collected organs were measured to the nearest 0.001 g. On the breast of all individuals, coloration was measured visually on a scale spanning from − 8 for white to 2 for dark reddish and the size of black feather spots were measured to the nearest 0.1 mm. Methods of assessing these two plumage traits are reliable (Roulin 2004b). Sex was identified with molecular methods.

### Assessment of melanin pigments

The ethanol contained in internal organs was evaporated in a desiccator and the dried organs (20–25 mg) was homogenized at a concentration of 20 mg/ml water using Wheaton Ten-Broeck tissue glass homogenizer (Wheaton, Tokyo). For the eyes and skin, homogenization was performed at a concentration of 10 mg/ml. Aliquots of 100 µl (2 mg dried tissue or 1 mg for the eyes and skin) were subjected to acid hydrolysis followed by alkaline H_2_O_2_ oxidation (Ito et al. 2018a), and hydroiodic acid hydrolysis (Wakamatsu et al. 2002). For the acid hydrolysis, water suspensions of samples in 10 ml screw-capped test tubes were evaporated to dryness in a desiccator. The samples were mixed with 0.5 ml 6 M HCl and heated at 110 °C for 16 h. The melanin suspensions were mixed with 1 ml water and centrifuged at 15,000 g for 10 min after which the supernatants were removed, and the pellets of melanin were washed once with 1 ml water by centrifugation. The remaining melanin was subjected to the alkaline H_2_O_2_ oxidation (Ito et al. 2011). The acid hydrolysis removes protein and low-molecular-weight components in tissue samples, and thus gives more simplified HPLC chromatograms compared with conventional H_2_O_2_ oxidation. Melanin is a stable molecule and hence freezing samples should not have altered its concentration.

### Statistics

All statistical analyses were performed with the software JMP13. Tests are two-tailed and p values smaller than 0.05 significant. The measured melanin pigments were Box–Cox transformed to obtain a normal distribution of the data. Because the measured melanin in the skin, heart, and trachea was related to the size of the sample (in grams; only aliquots of the organs were collected) (Table 1), for each organ, we extracted the residuals from a regression of the Box–Cox transformed eumelanin (or pheomelanin) on sample size. In this way, we obtained a measure about the concentration of melanin per unit of organ. Means are quote ± SE.

**Table 1:** Pearson’s correlations between the Box–Cox transformed eumelanin and pheomelanin content in organs and the amount of collected organs (in grams) in the barn owl

	Eumelanin	Pheomelanin
Skin	***r* = − 0.46, *n* = 31, *p* = 0.008**	*r* = − 0.24, *n* = 31, *p* = 0.19
Pectoral muscle	*r* = -0.35, *n* = 30, *p* = 0.06	*r* = 0.06, *n* = 30, p = 0.76
Eye	*r* = -0.12, *n* = 27, *p* = 0.56	*r* = -0.16, *n* = 27, *p* = 0.42
Heart	***r* = -0.37, *n* = 31, *p* = 0.04**	*r* = -0.15, *n* = 31, *p* = 0.43
Liver	*r* = -0.16, *n* = 31, *p* = 0.38	*r* = -0.13, *n* = 31, *p* = 0.47
Trachea	*r* = 0.02, *n* = 30, *p* = 0.93	***r* = 0.53, *n* = 30, *p* = 0.003**
Uropygial gland	*r* = -0.22, *n* = 29, *p* = 0.24	*r* = 0.18, *n* = 29, *p* = 0.35

## Results

### Melanin contents

We estimated the contents of eumelanin and pheomelanin as pyrrole-2,3,5-tricarboxylic acid (PTCA) and 4-amino-3-hydroxyphenylalanine (4-AHP) values which are the degradation products of eumelanin and pheomelanin, respectively (Ito et al. 2011; Wakamatsu et al. 2002). The concentration (ng/mg) of PTCA and 4-AHP was much higher in the eyes (457 ± 51.4 and 42.2 ± 5.19) than in the skin (5.60 ± 0.45 and 3.30 ± 0.35), liver (3.86 ± 0.22 and 6.09 ± 0.46), muscle (3.29 ± 0.28 and 6.15 ± 0.58), trachea (2.51 ± 0.20 and 1.68 ± 0.21), heart (2.15 ± 0.12 and 11.4 ± 0.72) and the uropygial gland (0.95 ± 0.07 and 0.66 ± 0.09), respectively. By considering Box–Cox transformed melanin contents, we found that except for the eyes, if one organ had more eumelanin content than another, it does not necessarily also have more pheomelanin, suggesting that the pattern is not driven simply by increased overall melanin production (Table 2). The organs with more eumelanin than pheomelanin are the eyes (paired *t*-test: *t*_26_ = 65.45, *p* < 0.0001), skin (*t*_30_ = 13.99, *p* < 0.0001), trachea (*t*_29_ = 8.43, *p* < 0.0001) and uropygial gland (*t*_28_ = 2.34, *p* = 0.027). The organs with more pheomelanin than eumelanin are the pectoral muscles (*t*_30_ = 14.95, *p* < 0.0001), heart (*t*_30_ = 19.04, *p* < 0.0001) and liver (*t*_30_ = 14.66, *p* < 0.0001).

**Table 2:** Paired *t*-tests comparing the eumelanin (A) or pheomelanin (B) contents between organs in the barn owl

A. Eumelanin	Skin	Pectoral muscle	Eye	Heart	Liver	Trachea
Pectoral muscle	*t* = 8.17, *p* < 0.0001					
Eye	*t* = − 60.63, *p* < 0.0001	*t* = − 60.80, *p* < 0.0001				
Heart	*t* = 15.35, *p* < 0.0001	*t* = 10.11, *p* < 0.0001	*t* = 60.82, *p* < 0.0001			
Liver	*t* = 3.43, *p* = 0.002	*t* = − 6.83, *p* < 0.0001	*t* = 60.74, *p* < 0.0001	*t* = − 21.24, *p* < 0.0001		
Trachea	*t* = 11.31, *p* < 0.0001	*t* = 4.95, *p* < 0.0001	*t* = 60.82, *p* < 0.0001	*t* = − 5.67, *p* < 0.0001	*t* = 13.02, p < 0.0001	
Uropygial gland	*t* = 18.42, *p* < 0.0001	*t* = 16.06, *p* < 0.0001	*t* = 60.89, *p* < 0.0001	*t* = 60.89, *p* < 0.0001	*t* = 11.39, p < 0.0001	*t* = 29.69, *p* < 0.0001
B. Pheomelanin	Skin	Pectoral muscle	Eye	Heart	Liver	Trachea
Pectoral muscle	*t* = − 14.89, *p* < 0.0001					
Eye	*t* = − 29.02, *p* < 0.0001	*t* = − 27.03, *p* < 0.0001				
Heart	*t* = − 16.58, *p* < 0.0001	*t* = − 6.71, *p* < 0.0001	*t* = 25.81, *p* < 0.0001			
Liver	*t* = − 14.59, *p* < 0.0001	*t* = − 0.66, *p* = 0.52	*t* = 27.78, *p* < 0.0001	*t* = 7.17, *p* < 0.0001		
Trachea	*t* = 5.65, *p* < 0.0001	*t* = 24.26, *p* < 0.0001	*t* = 29.65, *p* < 0.0001	*t* = 22.60, *p* < 0.0001	*t* = 25.38, *p* < 0.0001	
Uropygial gland	*t* = 7.67, *p* < 0.0001	*t* = 25.35, *p* < 0.0001	*t* = 30.11, *p* < 0.0001	*t* = 22.43, *p* < 0.0001	*t* = 24.95, *p* < 0.0001	*t* = 3.15, *p* = 0.0045

All degrees of freedom are 23 and tests were performed on the Box–Cox transformed data. Cells are in gray when the melanin content of the organ indicated in the top row is higher than the melanin content of the organ indicated in the first column. For example, the skin has more eumelanin than the pectoral muscle but less than the eyes

### Comparing melanin contents between organs

**Fig. 2:**
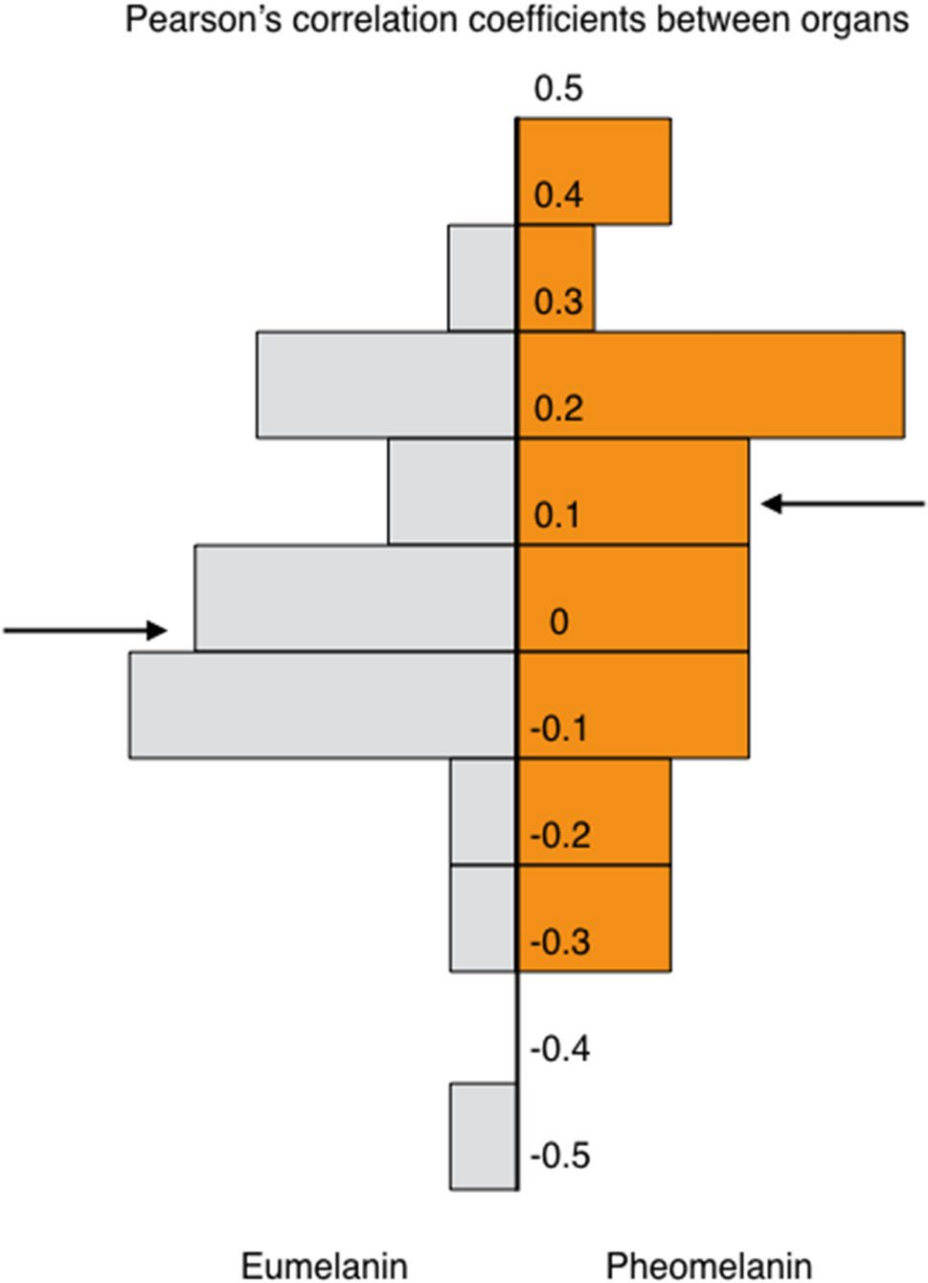
Frequency distribution of 21 pairwise Pearson’s correlations between the Box–Cox transformed eumelanin content in the different organs and 21 pairwise Pearson’s correlations between the Box–Cox transformed pheomelanin content in the different organs. For example, we calculated the correlation between the eumelanin content found in the eyes with the eumelanin content in the six other organs and tissues. Before performing these correlations, we first removed the variation explained by the amount of collected organ (in grams) to measure melanin content by extracting the residuals of the regression of melanin content on sample size. The arrows indicate the mean Pearson’s correlation for eumelanin (0.03) and pheomelanin (0.09)

The only significant within-individual correlation between the melanin detected in pairs of organs was between pectoral muscle and trachea for eumelanin (*r* = − 0.41, *n* = 29, *p* = 0.026) and for pheomelanin between skin and trachea (*r* = 0.45, *n* = 30, *p* = 0.01), between pectoral muscle and liver (*r* = 0.36, *n* = 30, *p* = 0.049) and between heart and liver (*r* = 0.41, *n* = 31, *p* = 0.02) (Fig. 2). The magnitude of the Pearson’s correlations between organs in the eumelanin content was not significantly different than the magnitude of the correlations between organs in the pheomelanin content (paired *t*-test: *t*_20_ = 1.40, *p* = 0.18; mean Pearson’s correlation coefficient ± SE: 0.004 ± 0.04 vs. 0.09 ± 0.05).

### Within-organ correlation between eumelanin and pheomelanin contents

**Fig. 3:**
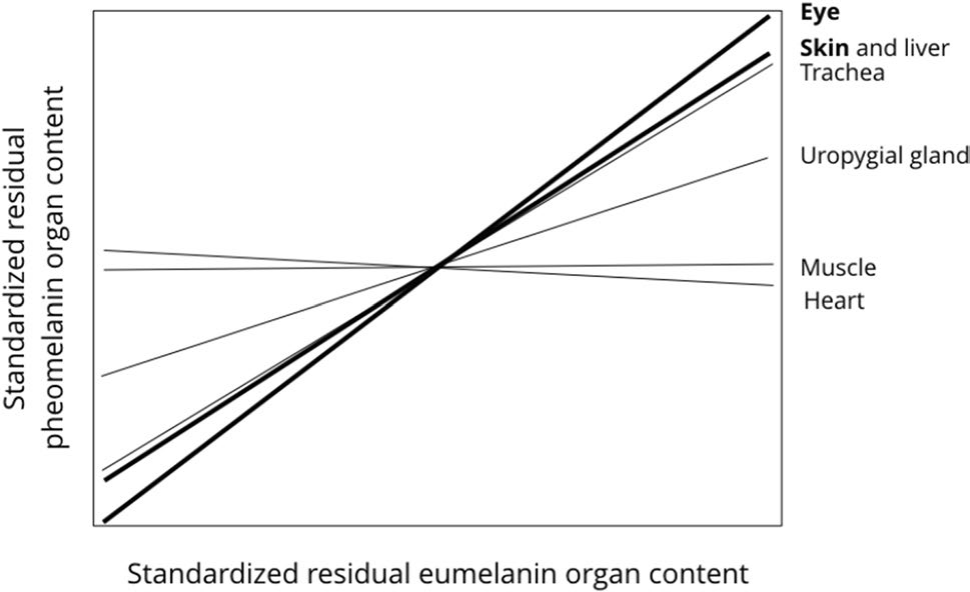
Relationship between the standardized eumelanin and pheomelanin contents in seven organs in the barn owl. We first removed the variation explained by the amount of collected organ (in grams) to measure melanin content and hence extracted the residuals of the regression of melanin content on sample size. Then, for each organ, we standardized these values ([value – mean]/SD) to compare the different organs. Significant relationships are written and drawn in bold

The eumelanin content was significantly correlated with the pheomelanin content in the eyes and skin and close to significance in the liver (Table 3; Fig. 3). In the heart and pectoral muscle, there was no sign of any relationship (Table 3).

**Table 3:** Pairwise Pearson’s correlations between the eumelanin and pheomelanin contents inside organs

	Correlation between eumelanin and pheomelanin
Skin	*r* = 0.34, *n* = 31, *p* = 0.059
Pectoral muscle	*r* = 0.003, *n* = 30, *p* = 0.99
Eye	***r* = 0.40, *n* = 27, *p* = 0.036**
Heart	*r* = -0.03, *n* = 31, *p* = 0.88
Liver	*r* = 0.34, *n* = 31, *p* = 0.06
Trachea	*r* = 0.32, *n* = 30, *p* = 0.08
Uropygial gland	*r* = 0.17, *n* = 29, *p* = 0.37

For each value, we first removed the variation explained by the amount of collected organ (in grams) to measure melanin content (see Table 1) by extracting the residuals of the regression of melanin content on sample size

### Sexual dimorphism in melanin content

Residual Box–Cox transformed eumelanin content was not sexually dimorphic for any of the organs (*p* values > 0.37). Residual Box–Cox transformed pheomelanin content was significantly sexually dimorphic only in the eyes (Student’s t-test: *t*_24_ = 3.12, *p* = 0.005; for the other organs *p* values > 0.08). Females had more pheomelanin than males in their eyes.

### Melanin content and plumage traits

**Fig. 4:**
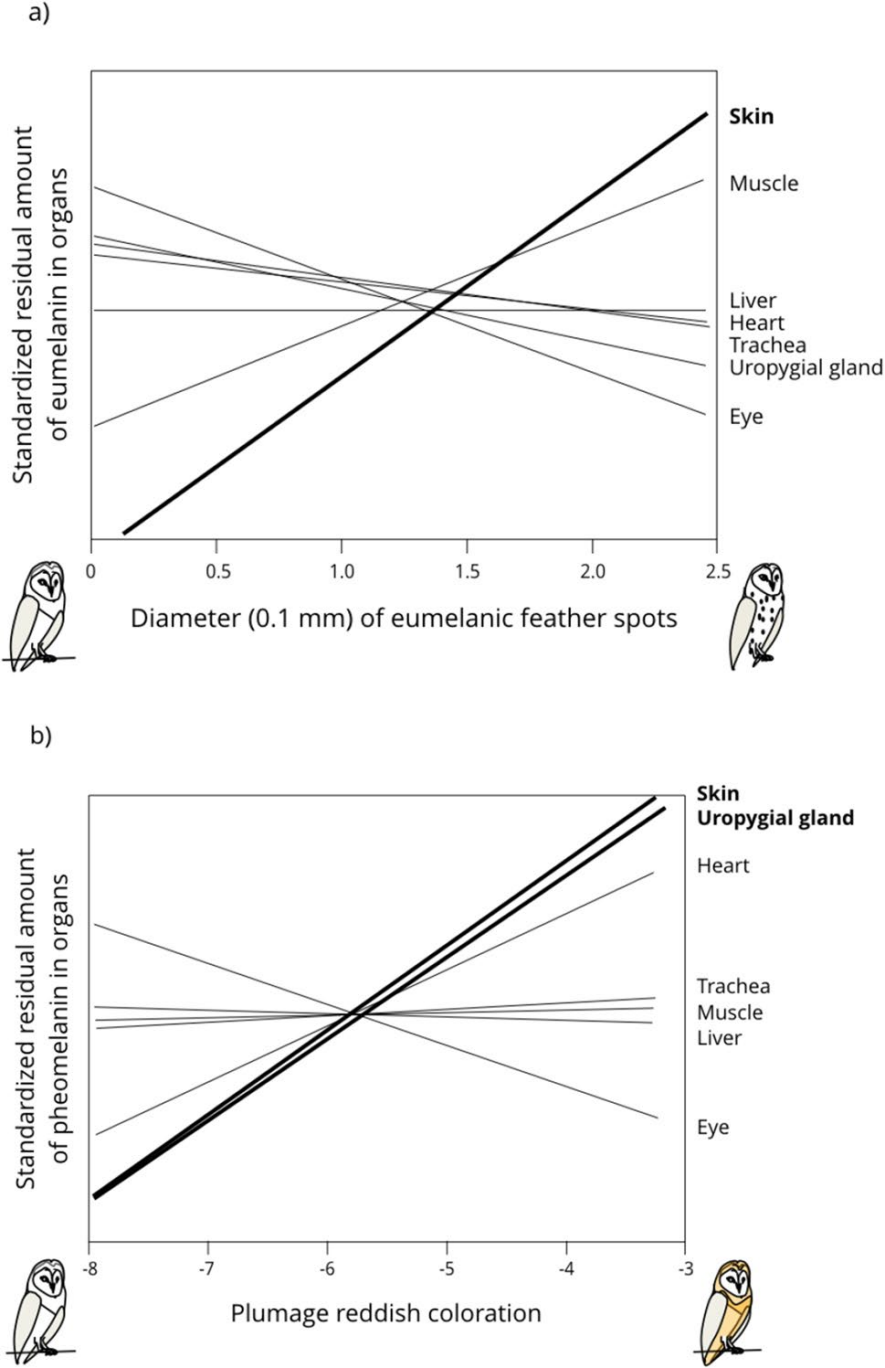
Relationship between plumage traits and the eumelanin (**a**) and pheomelanin contents (**b**) inside seven organs in the barn owl. We first removed the variation explained by the amount of collected organ (in grams) to measure melanin content by extracting the residuals of the regression of melanin content on sample size. Then, for each organ we standardized these values ([value—mean]/SD) to compare the different organs. Significant relationships are written and drawn in bold

**Fig. 5:**
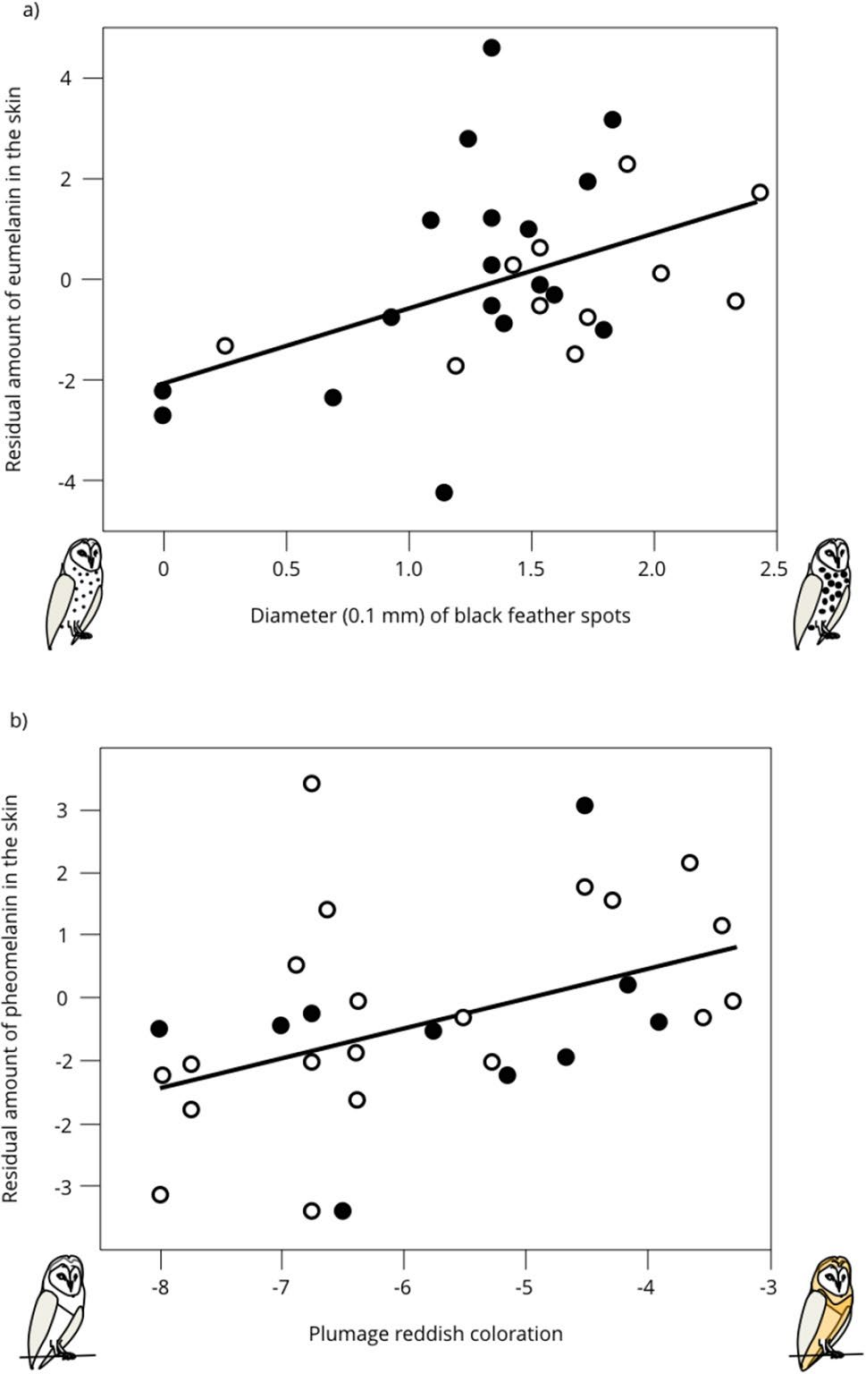
Eumelanin (**a**) and pheomelanin contents (b) in the skin of barn owls in relation to the size of black feather spots (**a**) and reddish coloration (**b**). Open circles are for females and closed circles for males. We first removed the variation explained by the amount of collected organ (in grams) to measure melanin content by extracting the residuals of the regression of melanin content on sample size. Regression lines are drawn for illustrative purposes

Figure 4a shows regression lines between the eumelanin content in the seven organs and the size of black eumelanic feather spots. Only the relationship between eumelanin in the skin and spot size was significantly positive (Pearson’s correlation on residual Box–Cox transformed residual eumelanin content: *r* = 0.46, *n* = 29, *p* = 0.013; Fig. 5a).

Figure 4b shows regression lines between the pheomelanin content inside organs and reddish brown coloration. The relationship between pheomelanin content of the skin (*r* = 0.40, *n* = 31, *p* = 0.026; Fig. 5b) and uropygial gland (*r* = 0.38, *n* = 29, *p* = 0.044) was significantly positive.

## Discussion

One of the major goals of the present study was to examine whether melanin-based coloration of the external body surface is related to the amount of melanin packed inside organs. In such a case, the implication would be that plumage coloration could reflect how much organs are protected from external sources of stress by melanin pigments. For instance, if coloration is related to skin melanin content, it could indicate that darker-colored birds are more resistant to parasites because they have more melanin in the integument as a first line of parasitic defense.

### How does melanin content of internal organs and skin associate with one another and what function melanin might serve in these tissues?

Our descriptive study in the barn owl shows that plumage coloration is mainly associated with melanin content measured on the external body surface (i.e., skin and uropygial gland) but less so with the melanin content measured in internal organs. To the best of our knowledge, few other studies have compared the melanin contents of multiple organs and body parts (e.g., Franco-Belussi et al. 2013). A study in European humans showed that coloration of the eyes, skin, and hair was correlated (Pearson’s correlations, 0.47 > r > 0.40): human populations with darker hair have darker skin and eyes (Candille et al. 2012). The strength of these correlations is relatively low because natural and sexual selection differentially affect skin, hair, and eye coloration so that geographic variation in skin pigmentation is stronger than geographic variation in hair and iris color (most human populations have dark hair and irises) (Parra 2007; the intensity of coloration is related to melanin production, Wakamatsu and Ito 2023). If some ecological factors such as climate can trigger the evolution of dark pigmentation on several body parts, as shown in lemurs (Rakotonirina et al. 2017), a key question is whether within a population, individuals that display darker hair, feathers, or cuticle have also a darker skin, eyes and whether their organs also produce more melanin pigments. This is not granted, as melanin can protect internal organs from heat stress, whereas it has been shown in the barn owl that individuals are paler in regions where temperatures are higher (Romano et al. 2019).

Across barn owls, the melanin content found in one organ was not or weakly correlated with the melanin content found in another organ. This suggests that the production of melanin in different parts of the body is independently regulated and could depend on the current need for melanin. Indeed, melanin production and concentration in internal organs are largely dependent on the surrounding environment (Guo et al. 2023). For example, melanin production in the eyes is regulated by light exposure (Ito et al. 2018b) and the body’s circadian rhythm (Bery et al. 2022), while melanin production in other organs may depend on the hormones they secrete (Guo et al. 2023), or other environmental factors (Slominski et al. 2022). As such, the production of melanin in internal organs is dynamic and can be affected by different environmental changes (Franco-Belussi et al. 2016).

Then, will the impacts of stress on melanin be seen more rapidly in organs than in plumage color? It is not possible to answer this question definitively, as the impacts of stress on melanin will vary depending on the species involved. It is possible, but probably not likely that the impacts of stress on melanin production may be seen more rapidly in organs than plumage because organs have a shorter growth period than plumage. However, this will depend on the type of stress and the physiological responses of the species. The effects of stress on melanin production may become evident in plumage color before organs if the responses of the species take longer to manifest in the plumage. Stress and many other environmental factors can affect melanin levels, but typically the impacts of stress on organs would not be seen as dramatically or as quickly as changes in plumage color. Stress can cause various types of physiological responses in organisms including changes in hormone levels, and the impacts of this on organ melanin content could be shorter to manifest than on the change in plumage color. Given that feather coloration is static (aside from color changes due to wear), could the time since molt influence whether a correlation between color and internal melanin is present?

It is generally accepted that the correlation between feather color and internal melanin is largely static over time. This correlation is mainly related to the genetic makeup of the individual bird and does not change significantly because of molt or the amount of time that has passed since the molt. However, it is possible that the correlation could slightly weaken over time if the feathers begin to wear due to aging, environmental conditions, or other factors. The time since molt could influence the correlation between color and internal melanin in some species of birds. In species that tend to darken their feathers over time, such as many raptors, this increase in color might be associated with increased melanin production also in internal organs.

Overall, although plumage color may not provide real-time information about dynamic internal melanin levels, it still serves as a reliable signal of the individual's condition and quality at the time of molting, and can provide insights into past environmental conditions and long-term melanin patterns. In summary, despite the molting cycle and the dynamic nature of melanin in the organ, the pattern and concentration of melanin developed during molting can persist for long periods, so static plumage color could remain informative of the content of melanin in organs. Preening behavior also contributes to maintaining the integrity of feather color. Additionally, correlations and patterns may exist between internal melanin levels and feather color, although they do not reflect real-time changes. Static plumage color could therefore convey information about an individual's condition and provide insight into past environmental conditions and long-term melanin patterns.

### How do plumage color/traits relate to internal melanin content?

The eumelanin and pheomelanin contents were positively correlated in the eyes and skin (but also in the liver and trachea) but not in the uropygial gland, pectoral muscle, and heart. These results show that the production of eumelanin can be decoupled from the production of pheomelanin, which can be also the case for plumage traits. Although reddish barn owls usually display more and larger black feather spots than white barn owls, the correlation between the degree of plumage reddishness and the size of black spots is not perfect. We can indeed observe white birds with many large spots (Roulin 2016).

In the barn owl, the eyes are black and unsurprisingly rich in eumelanin and to a lower extent in pheomelanin. A major function of melanin is to protect the eyes against antioxidants and UV light (Peles and Simon 2012). Similarly, the skin is the second tissue with a relatively high concentration of eumelanin. This is in line with the fact that eumelanin is one of the first lines of defense against numerous aggressions from the environment. The finding that the ectoparasitic fly *Carnus hemapterus* lay fewer eggs if feeding on heavily spotted than lightly spotted nestling barn owls (Roulin et al. 2001) could be indeed explained by a higher quantity of eumelanin in the skin in heavily than lightly spotted barn owls. The presence of eumelanin in the skin may prevent parasites from extracting all the necessary resources from their host to produce eggs. Pheomelanin may have other functions, particularly in muscles (e.g., pectoral muscle and heart) and liver, where we detected high concentrations of this pigment, which could also partly explain their reddish coloration. It therefore seems that to fully understand the function of eumelanin and pheomelanin, we should simultaneously consider the role played by melanin-based coloration of the external body surface and the biophysical function of melanin on the external body surface and inside the body.

To conclude this paper, we believe that more data are required about the melanin content of internal organs in birds to understand the full signaling function of melanin-based coloration. The present study is a first attempt in this direction.

## Conclusion

In birds, melanin pigments are distributed internally and externally. Internal melanin is the pigment that governs the color of internal tissues and organs, while external melanin is the pigment that appears in feathers and skin on the surface of the body. Although some studies are currently underway on the relationship between internal and external melanin, it is not yet fully elucidated. Here is a rundown of what we know and what we do not know:

We know that (1) higher levels of internal melanin tend to darken body tissues and organs (Franco-Belussi et al. 2016, 2017); (2) The higher the level of external melanin, the darker the surface of the body tends to be (Del Bino et al. 2015, 2022; Wakamatsu and Ito 2021); (3) The amount of internal and external melanin can affect the reproductive and immune systems of birds (Jaquin et al. 2011; Dubey and Roulin 2014); (4) External environmental factors (light irradiation, nutritional status, etc.) affect the production of internal and external melanin (Costin and Hearing 2007; Mauldin et al. 2016; Boo 2020).

We do not know (1) The specific relationship and control mechanism between internal and external melanin; (2) Studies on the interactions and correlations between internal and external melanin are limited and a better understanding is needed; (3) Internal melanin and external melanin may influence each other, but the specific mechanism is unknown; (4) The relationship between internal and external melanin may differ between individual birds and species. Research on these unexplained points is underway and is expected to provide important knowledge about color formation and evolution in birds.
